# Behavioural analysis of referral and uptake of care pathways for rare tumour risk syndromes: A cross-cultural mixed-methods study protocol within the PREVENTABLE project

**DOI:** 10.12688/openreseurope.19954.2

**Published:** 2026-07-06

**Authors:** Ana Machado, Maiara Moreto, Isabel Fernandes, Ana Maria Rodrigues, Bárbara Peleteiro, Joan Brunet, Judith Balmaña, Claude Houdayer, Jean-Christophe Thery, Janneke H.M. Schuurs-Hoeijmakers, Stefan Aretz, Cathrine Bjorvatn, Liliana Sousa, Sara Pereira, Carla Oliveira, PREVENTABLE Clinical Research Team, Marta M. Marques

**Affiliations:** 1 Comprehensive Health Research Centre (CHRC), NOVA Medical School, NOVA University of Lisbon, Lisbon, Portugal; 2 EpiDoc Unit, NOVA Medical School, NOVA University of Lisbon, Lisbon, Portugal; 3 Oncology Department, Hospital CUF Descobertas, Lisbon, Portugal; 4 Rheumatology Department, Hospital Egas Moniz, Centro Hospitalar Lisboa Ocidental, Lisbon, Portugal; 5 Rheumatology Department, Hospital dos Lusíadas, Lisbon, Portugal; 6 Hospital Epidemiology Center, Unidade Local de Saúde São João, Porto, Portugal; 7 Faculty of Medicine, University of Porto, Porto, Portugal; 8 EPI Unit, Institute of Public Health, University of Porto, Porto, Portugal; 9 Associate Laboratory for Integrative and Translational Research in Population Health (ITR), University of Porto, Porto, Portugal; 10 Catalan Institute of Oncology, Barcelona-Girona, Spain; 11 Girona Biomedical Research Institute-IDIBGI, Girona, Spain; 12 ERN GENTURIS - European Reference Network on Genetic Tumour Risk Syndromes, Nijmegen, The Netherlands; 13 Medical Oncology Department, Vall d’Hebron Barcelona Hospital Campus, Barcelona, Spain; 14 Hereditary Cancer Genetics Group, VHIO - Vall d’Hebron Institute of Oncology, Barcelona, Spain; 15 Department of Genetics, Univ Rouen Normandie, INSERM U1245, FHU-G4 Génomique and CHU Rouen, Rouen, France; 16 Department of Medical Oncology, Center Henri Becquerel, University of Medicine of Rouen, Rouen, France; 17 Department of Human Genetics, Expert Center for PHTS, Radboud University Medical Center, Nijmegen, The Netherlands; 18 Institute of Human Genetics, Medical Faculty, University of Bonn, Bonn, Germany; 19 National Centre for Hereditary Tumour Syndromes, University Hospital Bonn, Bonn, Germany; 20 Department of Medical Genetics, Haukeland University Hospital, Bergen, Norway; 21 Universidade do Porto Instituto de Investigacao e Inovacao em Saude, Porto, Portugal; 22 University of Porto Institute of Molecular Pathology and Immunology, Porto, Portugal; 23 New University of Lisbon Comprehensive Health Research Centre National School of Public Health, Lisbon, Portugal

**Keywords:** barriers; facilitators; communication; behaviour change; decision-making; hereditary cancer; rare diseases

## Abstract

**Background:**

Individuals with rare tumour risk syndromes (RTRS) navigate complex care pathways, including early diagnosis, monitoring, and tailored treatments, all requiring shared decision-making with their clinical team. Nevertheless, behavioural factors influencing this decision-making process and the best communication practices are unknown.

**Objective:**

This study aims to identify the behavioural factors (i.e., barriers and facilitators) influencing i. the recommendation of RTRS-care pathways by members of clinical teams, ii. the uptake of the care pathways by patients with RTRS, and iii. The factors experienced by patients’ social networks. This study is part of PREVENTABLE, an EU-Horizon funded project, aiming to assess the clinical, social and economic impact of RTRS-care pathways.

**Methods:**

A cross-cultural mixed-methods study with a sequential explanatory design will be conducted, consisting of i. a web-based survey study (quantitative phase), and ii. a qualitative study conducted through focus groups (qualitative phase). First, two international web-based surveys, following the COM-B model of behavioural change, will be developed aiming to investigate the barriers and facilitators to i) the recommendations of RTRS-care pathways by 100 members of clinical teams managing these syndromes, and ii) the uptake of the recommended care pathways by 290 patients with RTRS. The results will inform the focus groups phase aiming to provide an in-depth exploration of the main factors affecting the recommendation and adoption of RTRS-care pathways. The study will involve six countries, i.e., France, Germany, Netherlands, Norway, Spain, and Portugal. Both qualitative and quantitative data will first be analyzed separately, and then merged through data triangulation for final interpretation of the results.

**Conclusion:**

This will be the first study exploring the behavioural factors influencing the recommendation and uptake of RTRS-care pathways. This study will guide the development of guidelines for the communication and recommendation of optimized care pathways for RTRS, which might enhance the referral and uptake of RTRS preventive strategies and inform the best care for RTRS.

## Plan language summary

People with rare tumour risk syndromes (RTRS) are confronted with a lot of new information and complex personal decisions about their care. To improve the care these individuals receive, it is essential to improve communication and understand which factors are making it easier and/or more difficult for them to decide on the different care options. This study will explore the behavioural factors that have been influencing the recommendation and uptake of care options for RTRS by clinical teams, patients with RTRS and their social networks. To do this, we will conduct online surveys and group discussions with clinical teams, patients with RTRS and their social networks. This study is part of a larger EU-funded project, the PREVENTABLE project ( https://preventable.eu/), and is being conducted in six countries – Portugal, Spain, France, Germany, Netherlands and Norway. Understanding these behavioural factors will help us developing strategies to improve the communication and recommendation of care to these patients, possibly leading to a better care for them.

## Background

Rare tumour risk syndromes (RTRS) are a set of genetically determined rare diseases, in which the lifetime risk of developing various types of cancer is up to 100%. Since these syndromes follow an autosomal dominant inheritance, patients have a 50% chance of transmitting the disease to their offspring. ^1, 2^ When undiagnosed or not surveilled, people with RTRS develop particularly aggressive cancers, which result in premature death or severely impact theirs and their family health. ^3– 5^ Nevertheless, cancers in people with RTRS can be prevented and survival rates maximized if: i) asymptomatic carriers are intensively surveilled for RTRS cancer-prone organs, ii) cancer-prone organs are surgically removed prior to disease development, if adequate, and iii) very small cancerous or pre-cancerous lesions are removed or treated. ^6– 9^ RTRS constitute, therefore, an unique context for cancer prevention, early detection and treatment with curative intent.

When diagnosed, individuals with RTRS have not only to understand their diagnosis and its implications, but they are also presented with different care pathways — i.e., sequences of care options that involve multiple decisions, such as primary prevention strategies (e.g., risk-reducing surgeries and lifestyle modifications), surveillance options (e.g., more frequent screenings) and treatment for cancer or premalignant conditions. ^2, 10^ Furthermore, beyond managing their own diagnosis, they are also responsible for communicating the results to their family, and for informing at-risk family members about their genetic risk, leading to a cascade genetic testing. ^2, 10^ These individuals are therefore confronted with complex and personal decisions, which often lead to uncertainty, doubts, distress and feelings of guilt. ^11– 14^ In this context, a clear, empathetic, and evidence-based communication of risks, treatment strategies and surveillance plan is essential for quality decision-making in individuals with RTRS. ^10^ The vital role of individuals’ social support systems in helping managing ongoing medical monitoring and adhering to recommended care must also be acknowledged. ^2^ Further, any variations in healthcare professionals’ guidelines and recommendations for RTRS across countries can influence the decision-making process. Guidelines for RTRS are scarce and rarely address the personal needs or actions of people with RTRS, such as psychosocial support or lifestyle behaviours, posing further challenges to healthcare professionals. ^2^ Furthermore, gaps still persist in understanding the various factors influencing decision-making in RTRS. These include the preferences of individuals with RTRS, members of clinical teams recommendations, and the best practices for communication and emotional support in RTRS care. ^10^ In order to improve care in RTRS, it is therefore crucial to overcome the hurdles in communication between these individuals and their clinical teams. ^15^ Conducting research using behavioural science might help facilitating informed decisions in this context. ^2^ The behavioural analysis refers to the systematic examination of factors that influence two specific target behaviours: (1) the referral and recommendation of RTRS care pathways by members of clinical teams, and (2) the uptake of recommended care pathways by people with RTRS. Our behavioural analysis explores the psychological, social, and environmental factors that may enable or hinder these behaviours.

In this context, one of the most widely used behaviour science models that was developed to inform and guide practice is the Capability-Opportunity-Motivation-Behaviour (COM-B), which provides comprehensive understanding of the behavioural factors influencing individuals’ decision-making process. ^16– 20^ According to this model, for any given behaviour to take place, the individual must have the capability, opportunity and motivation to engage in that behaviour. ^21, 22^ Capability refers to both physical capability, involving skills, strength, and stamina; or psychological capability, involving knowledge and cognitive ability to perform the behaviour. ^21, 22^ Opportunity refers to external factors and includes physical opportunity, which refers to environmental aspects such as resources and time; and social opportunity, which relates to cultural influences, including family and social networks. ^21, 22^ Motivation includes reflective motivation (e.g., conscious planning) and automatic motivation (e.g., emotions and impulses). ^21, 22^ Furthermore, the COM-B model captures both internal (psychological capability and motivation) and external (physical and social opportunities) determinants of behaviour, enabling to systematically identify barriers and facilitators in health contexts, ^20^ such as the referral and recommendation of care pathways for RTRS. Its use provides a systematic understanding of the factors influencing behaviour, and offers a structured approach for the development of tailored interventions by mapping the types of strategies likely to influence each construct effectively. ^23^ For the qualitative component, we will additionally use the Theoretical Domains Framework (TDF) as a complementary framework to support more in-depth exploration of the determinants influencing behaviours. ^24^

The PREVENTABLE consortium – an EU-Horizon funded project aiming to assess the clinical, social and economic impact of applying preventive care pathways for families with RTRS. The PREVENTABLE project aims to address the gaps in the risk communication and decision-making process by focusing on two target behaviours: i. referral and recommendation to care pathways by members of clinical teams and ii. uptake of recommended care by patients. The study investigates the behavioural determinants underlying each of these behaviours. These determinants include individual, social, and environmental factors (e.g., knowledge, beliefs, social support, clinical setting) that may act as barriers or facilitators for each distinct behaviour. By examining these determinants, the study aims to clarify how they influence both the members of clinical teams’ recommendations and the patients’ uptake of care pathways for RTRS.

## Objectives

This study protocol describes a mixed-methods study that aims to identify the behavioural factors influencing the recommendation and uptake of care pathways for RTRS across European countries involved in the PREVENTABLE consortium – i.e., Portugal, Spain, France, Germany, the Netherlands, and Norway.

Specifically, we aim to:

1. Identify the barriers and facilitators influencing the recommendation and referral of care pathways for RTRS by members of clinical teams managing these syndromes.
2. Identify the barriers and facilitators influencing the uptake of care pathways for RTRS by individuals with these syndromes.
3. Identify the barriers and facilitators influencing how the social network of individuals with RTRS provide support for, engage in, and influence the uptake of the recommended care pathways.

## Protocol

### Design

A cross-sectional mixed-methods study with a sequential explanatory design (QUAN → QUAL) ^24, 25^ will be conducted, encompassing two steps: 1) a web-based survey study (quantitative phase), and 2) a qualitative study conducted through focus groups (qualitative phase). In this methodology, qualitative methods are used to provide breath of understanding to the quantitative results by exploring participants’ experiencies, perceptions and contextual factors related to barriers and enablers identified in the survey. This approach maximizing the strengths and minimizing the weaknesses of each type of data. ^24, 25^ Study registration is available at Open Science Framework: https://doi.org/10.17605/OSF.IO/KRQFA.

In step 1, two online anonymised surveys – one for members of clinical teams managing RTRS and one for individuals with RTRS are conducted. In step 2, to further elaborate on the findings from the surveys, we will conduct focus groups with: i. members of clinical teams who specialize in RTRS, ii. patients with RTRS and iii. Members of the social network of patients with RTRS. Members of the social network are only included in Step 2 as an additional source to examine how their involvement may affect patients’ decisions and support individuals with RTRS in navigating their care pathways.

The study is being conducted in six European countries that are part of the PREVENTABLE consortium, i.e., Portugal, Spain, France, Germany, Netherlands and Norway.

### Population

Seven RTRS will be included: Birt-Hogg-Dubé Syndrome (variants in *FLCN* gene), Familial Melanoma (variants in *CDKN2A* or *CDK4* genes), Hereditary Diffuse Gastric Cancer (variants in *CDH1* or *CTNNA1* genes), Li-Fraumeni Syndrome (variants in *TP53* gene), Peutz-Jeghers Syndrome (variants in *STK11* gene), PTEN Hamartoma Tumour Syndrome (variants in *PTEN* gene), Hereditary Leiomyomatosis and Renal Cell Carcinomas (variants in *FH* gene). These seven RTRS are part of the thematic groups 2 and 4 of the ERN Genturis.

The inclusion criteria for the study will be as follows: For members of clinical teams, eligible participants must: 1) be aged ≥18 years old, and 2) be members of clinical teams who have experience in providing recommendations of care to individuals with at least one of the RTRS listed above. For individuals with RTRS, participants must: 1) be aged ≥18 years old, and 2) be diagnosed with one of the RTRS listed above.

For the online surveys, we aim to include 100 healthcare professionals with experience with RTRS and 290 individuals with RTRS. A sample size calculation to obtain a representative sample of individuals with RTRS was performed based on the total number of RTRS cases identified within the PREVENTABLE consortium (n = 1177) and assuming a 95% confidence interval and 5% margin of error. For members of clinical teams, the sample size was determined based on the estimated number of healthcare professionals with expertise in the syndromes included in the PREVENTABLE project and the composition of the participating teams across countries.

For the focus groups, the same inclusion criteria used in the survey will be apply to members of clinical teams and individuals diagnosed with RTRS. In addition, this step will include a third stakeholder: members of the social network of individuals diagnosed with one of the RTRS listed above. All focus groups participants must be aged ^3^ 18 years old. Following stablished recommendations, approximately six participants per focus group will be included. ^26^ In each country of the consortium, three focus groups will be conducted (one per target group – i.e., members of clinical teams, individuals with RTRS, social networks). Hence, in total, 18 focus groups will be conducted, corresponding to a total of 108 participants, 36 participants per target group.

All participants in the survey or focus groups must be able to provide informed consent. Potential participants will be excluded if they are not fluent in Portuguese, Spanish, Catalan, French, German, Dutch or Norwegian. Additionally, for the focus groups, individuals with significant hearing or speaking impairments (e.g., deaf, mute) or other conditions that can impair verbal communication will be excluded.

### Development of the surveys

The surveys aim to explore the barriers and facilitators to i) the referral of the best care pathways for RTRS by members of clinical teams managing these syndromes, and ii) the uptake of the recommended care pathways by individuals with RTRS. The surveys were developed, in English, based on the COM-B model of behaviour change which attributes behaviour to three factors: physical and psychological capability (e.g., energy, knowledge), physical and social opportunity (e.g., access to care, social support), and reflective and automatic motivation (e.g., beliefs, associative learning). ^21^

The process of survey development consisted of 1) drafting the questions by the research team who has expertise in behavioural and social sciences and health sciences, 2) readjusting based on feedback from PREVENTABLE consortium members experts in RTRS, 3) pilot testing, and 4) refinement of surveys and translation to each PREVENTABLE language. The drafting of questions was based on a revision of the literature on RTRS, analysis of previous studies following the COM-B model, and the findings of a preliminary pilot survey conducted with 11 experts of PREVENTABLE, in which six barriers and six facilitators were identified ( https://osf.io/j9xmy). ^27^ The draft surveys were explored in two meetings with experts (one per survey) with the support of the PREVENTABLE consortium members and partners, who are experts in the field. Feedback on the survey for members of clinical teams was obtained in one meeting with the clinical partners of the consortium, which were given time to provide additional feedback by e-mail following the meeting. Feedback on the survey for patients was obtained in one meeting with the patients’ association EVITA, which was also given time to provide additional feedback by e-mail following the meeting. Following the adjustments based on experts’ feedback, the surveys were pilot tested. The survey for members of clinical teams was tested by two oncologists and one nurse. The survey for patients was pilot tested by four individuals with different rare syndromes. Following the adjustments provided by participants in the pilot testing, the final version of the surveys was reached and translated into Portuguese, Spanish, Catalan, French, German, Dutch and Norwegian, the official languages of the countries integrating the consortium. Translations were performed using the eTranslation service provided by the European Commission. ^28^ The translations were reviewed and adjusted for cultural adequacy by PREVENTABLE Clinical partners, who are bilingual experts with knowledge of both RTRS and the target healthcare context. This ensures that questions capture the intended concepts while being culturally and linguistically appropriate.

The final surveys are divided into three sections: 1) sociodemographic characteristics, 2) barriers and facilitators, and 3) ranking of barriers and facilitators. In total, the survey for members of clinical teams consists of 30 items and the one for patients includes 36 items. All the items are mandatory response. In the first section, information regarding age, sex (patients) or gender (healthcare professionals), country of residence, experience with RTRS (healthcare professionals), diagnosis (patients), clinical specialty (healthcare professionals), workplace (healthcare professionals) and level of education (patients) is collected. If a healthcare professional does not have experience with at least one of the RTRS included, or a patient does not have one of these RTRS as diagnosis, the survey will close. In Section 2, participants are asked to rate how much they agree/disagree with the role of different items related with capability, opportunity and motivation in their decisions/choices. Participants rate each item in a 7-points Likert scale ranging from 1-strongly disagree to 7-strongly agree. In the last section, participants are provided with a list of potential barriers and facilitators that could be associated with their referral options or decisions regarding care. Participants are asked to choose the three barriers and three facilitators that influence their decisions the most, and rank them from 1-the one with the most influence to 3-the one with least influence.

In the survey for healthcare professionals, psychological capability is assessed through items regarding knowledge, skills, and access to skills’ training. Physical opportunity is explored through items related to environmental context and costs. Social opportunity is explored through items related to social influences, social support and social norms. Reflective motivation is explored through professional role, personal beliefs and beliefs about the patient. Automatic motivation is explored through items related to habits and emotion regulation. The full survey is available at: https://doi.org/10.17605/OSF.IO/KRQFA.

In the survey for patients, physical capability is assessed through physical skills. Psychological capability is explored through items regarding knowledge, and memory, attention and decision processes. Physical opportunity is explored through items related to environmental context and costs. Social opportunity is assessed through social support and social influences. Reflective motivation is explored through beliefs and social influences. Automatic motivation is explored through items related to emotions. The full surveys are available at: https://doi.org/10.17605/OSF.IO/KRQFA.

### Development of the topic guides for the focus groups

Focus groups will aim to explore i) the factors that influence healthcare professionals in making referrals and recommendations of care for individuals with RTRS, ii) the factors that influence how individuals with RTRS make decisions about their care options, and iii) the challenges and supportive factors faced by those who provide support to individuals with RTRS.

Semi-structured interview guides, one per target group, will be used to ensure that the topics under investigation are covered in a consistent manner while allowing flexibility for new topics that are important for participants to emerge. The interview guides were developed based on the COM-B model of behaviour change, which categorizes behavioural factors into physical and psychological capability (e.g., energy, knowledge, beliefs), physical and social opportunity (e.g., access to care, social support, social norms) and reflective and automatic motivation (e.g., goals, planning emotions). ^21^ The process of topic guides development consisted of 1) drafting the discussion topics by the research team who has expertise in behavioural and social sciences and health sciences, 2) readjusting based on feedback from PREVENTABLE consortium members experts in RTRS, and 3) refinement of the topic guides and translation to each PREVENTABLE language. The drafting of the discussion topics was informed by the existing literature, ^26, 29^ the expertise of the members of the consortium, and the results of the surveys. A simulative case study is included to provide a basis for discussion. The interview guides for the focus groups were developed in English and translated into Portuguese, Spanish, Catalan, French, German, Dutch and Norwegian using the eTranslation service provided by the European Commission. ^28^ Clinical partners will review and adjust the translations for cultural adequacy. The topic guides are available at: https://doi.org/10.17605/OSF.IO/KRQFA.

### Recruitment

The survey for members of clinical teams was open for completion from 18 ^th^ June to 18 ^th^ October 2024. It was disseminated across Europe by ERN GENTURIS and PREVENTABLE’s clinicals partners through their networks. ERN GENTURIS disseminated the survey by e-mail by sending invitations to participate in the survey to their network of healthcare providers. PREVENTABLE’s clinical partners shared the survey by word of mouth and by e-mail. Clinical partners invited their colleagues to participate in the survey and to share it through their own networks of RTRS-experts. The survey was also sent by e-mail to nine professional societies linked to RTRS (e.g., Portuguese oncology society, Portuguese society of human genetics) with a request for dissemination through their networks. In addition, the survey was disseminated through PREVENTABLE’s social media platforms.

The survey for patients was open for completion from 18 ^th^ June to 24 ^th^ November 2024. It was disseminated across Europe by the patients’ association EVITA, other patients’ associations, ERN GENTURIS and PREVENTABLE’s clinical partners. The patients’ association EVITA disseminated the survey through their social media platforms, by word of mouth and by e-mail. EVITA contacted their partner patients’ associations across Europe and requested their support in sharing the survey with their network of patients. EVITA also engaged with their members to encourage them and their families to complete the survey. The survey was also sent by e-mail to 17 patients’ association related with RTRS with a request for dissemination through their networks. ERN GENTURIS disseminated the survey by inviting their network of patients’ associations to share the survey with their network of patients. PREVENTABLE’s clinical partners disseminated the survey by word of mouth and by e-mail. Clinical partners invited their patients with RTRS to participate in the survey and encouraged them to also involve their families in the study, both during scheduled appointments and by sending e-mails and invitation letters. In addition, the survey was disseminated through PREVENTABLE’s social media platforms.

Focus groups will be conducted between January and May 2025. Recruitment and facilitation of the focus groups will be ensured by each clinical site. A convenience sample will be used. The team at each clinical site will be responsible for identifying the eligible participants and provide them with an explanation about the study. Only the interested individuals will afterwards be contacted by the facilitator of the focus group at each clinical site, who will further explain the study, clarify any doubts and collect the informed consent. No information will be collected before the informed consent is provided. A verbal (recorded) informed consent will also be requested at the beginning of each focus group.

### Data collection

The anonymous surveys were made available in an online format using the LimeSurvey platform, which allows the completion of the surveys in any device with internet access (e.g., computer, mobile phone) and includes all the official languages of the countries integrating the consortium. By accessing the link to the online anonymous survey, potential participants were first presented with a welcome message with information about the study, inclusion/exclusion criteria and the informed consent. Participants were then required to provide their digital informed consent prior to any data collection. Only participants that provided the voluntary digital informed consent were able to continue to the survey. Participants were free to save the survey and continue answering later, in a more convenient time, and could also quit at any time by closing the survey. Cookies were used to avoid that the surveys were filled in more than once by the same person, i.e., a participant was not allowed to respond more than once from a single IP address, thus avoiding participants’ multiple participation in the LimeSurvey platform. To avoid questionnaire burden, the surveys were designed to take no longer than 10 minutes to be filled in.

Three focus groups will be conducted at each clinical site in Portugal, Spain, France, Germany, Netherlands and Norway, one for each of the target groups. The focus groups will be conducted in a quiet room at each clinical site or online (through the platform Microsoft Teams), depending on what is more convenient for the participants in each group. As recommended, each focus group will have approximately six participants, one facilitator and one assistant. ^26^ Each focus group is expected to last around 2 hours. All participants will sign an informed consent before participation. Semi-structured interview guides, one per target group, informed by the literature, the results of the surveys on the barriers and facilitators influencing the choice of the care pathways for RTRS, and the expertise of the members of the consortium, will be used to ensure that the topics under investigation are covered in a consistent manner while allowing flexibility for new topics that are important for participants to emerge. All focus groups will be recorded using audio recording devices. A verbal (recorded) informed consent will also be requested at the beginning of each focus group. The recordings will be transcribed verbatim using a dedicated software (i.e., the MAXQDA software) and translated into English language through sub-contracting the services of a translation company. Participants’ names will be anonymized. Information about age, sex (patients), gender (clinical teams, social network), diagnosis (patients), relationship (social network) and profession (clinical teams) of the included participants will be collected for sample characterization.

### Data analysis

Descriptive statistics will be used to characterize the sample, both in the surveys and in the focus groups.

The surveys will be analysed in an aggregated manner. Data analysis will include descriptive and inferential statistics. Descriptive statistics will be used to analyse each individual item. In addition, survey items will be grouped and analysed by the corresponding COM-B model domains, i.e., physical capability, psychological capability, physical opportunity, social opportunity, reflective motivation and automatic motivation. Within each COM-B domain, items will also be grouped by more granular categories of constructs. Inferential statistics will be used to allow comparisons between different COM-B domains and categories. Specifically, we will explore: (i) comparisons between the different care pathways, (ii) within-domain comparisons of more granular constructs, and (iii) comparisons of total scores across COM-B domains. In addition, we will examine whether the COM-B domain differ among syndromes, clinical specialties and countries.

The data from the focus groups will be analysed through thematic analysis. ^30^ The procedures proposed by Braun and Clarke will be followed. ^30^ Two researchers will be responsible for reading all the transcriptions and code them. Then, the research team will review the data coded under each theme/subtheme, contribute to the appropriate naming and definition of the themes, and produce the report. The criteria of credibility, transferability, dependability, and confirmability will be used to ensure rigor and trustworthiness. ^31^ The anonymous transcriptions will be analysed in an aggregated manner, per target group. Analysis of the transcripts will be conducted using the MAXQDA software. We will compare existing variations in how the COM-B domains are manifested between countries to address cross-country differences.

Quantitative and qualitative data will first be analysed separately, in a sequential process, and afterwards merged for integration of both methods in the final interpretation of the results. Findings from different stakeholder groups will be reported and interpreted independently. Triangulation of data within each stakeholder will be achieved using a narrative integration with a contiguous approach and integration through joint displays, allowing side-by-side presentation of the findings. This approach will facilitate the interpretations of the data by identifying convergences and divergences between the quantitative and qualitative findings.

### Privacy and data protection

All surveys are confidential and anonymous. No personal information is collected, hence, every data collected is irreversibly anonymous from the start. The anonymous surveys are built in and distributed through the LimeSurvey platform. This platform was chosen because it fully complies with the European Union’s general data protection regulation. To ensure full compliance, the German server location was defined. In addition, each participant’s anonymity is ensured by not using URLs, store internet protocol (IP) addresses nor requesting participants to register their identities, contact or email. By accessing the link to the online anonymous survey, potential participants are first presented with a welcome message with information about the study, inclusion/exclusion criteria and the informed consent. Participants are then required to provide their digital informed consent prior to any data collection. Only participants that provide the voluntary digital informed consent are able to continue to the survey. Participants are free to quit at any time by closing the survey.

Regarding focus groups data, personal information will be processed and secured in accordance with ethical principles and applicable international, European union and national legislation. All data subjects will confirm their consent by signing a free and fully informed consent form containing a detailed description in lay terms of the study and how data will be stored. All audio recordings collected within this study will be temporarily stored on a secured, password-protected folder in the central server from NOVA University of Lisbon, and immediately destroyed after the transcription and translation to English language.

Three databases will be generated in this study. Two databases are generated by the LimeSurvey platform, one per survey. In addition, characterization data from the focus groups will be anonymously stored, based on study ID, on a secured, anonymized, password-protected database. All anonymized databases will be stored in the central server from NOVA University of Lisbon. Only the research team involved in the study will have access to the anonymous databases. The anonymous databases will be kept for the duration of the project and 3 years beyond the project ending.

The privacy and confidentiality policies of the NOVA University of Lisbon will be followed. The study will comply with the ethical principles, data privacy protection and applicable Data Protection in all involved Institutions and Bodies, following the Regulation 2018/1725.

***Data collection.*** The protocol for data collection was made available as a pre-registry at Open Science Framework, and no alterations have been made since its initial publication. Data collection is currently underway.

### Dissemination

The results from this study will be made available to the scientific community and general population through their dissemination in peer-reviewed scientific publications, presentations in national and international conferences, and PREVENTABLE’s social media platforms.

## Conclusions

This will be the first study exploring the behavioural factors influencing the recommendation and uptake of care pathways for RTRS. By using a behaviour science model, this study will provide a systematic understanding of the main behavioural factors – i.e., barriers and facilitators – that should be targeted through behaviour change strategies to improve decision-making, referral and uptake of RTRS preventive care, among both patients with RTRS, their social networks, and members of clinical teams. The integration of the results of the focus groups and surveys will inform the development of guidelines on how the care pathways for RTRS should be communicated to patients using a person-centred approach, hence ensuring uptake. The guidelines will include sufficient information to allow improving the communication and understanding of RTRS prevention-related care pathways by the actors involved (members of clinical teams, patients, families), with a focus on risk communication, clinical outcomes and service facilitation.

## Ethics and consent

The study will be conducted in full conformance with the principles of the Declaration of Helsinki and Good Clinical Practice guidelines. It will comply with the ethical principles in all involved Institutions and Bodies, following the European Union Regulation 2018/1725. Written informed consent will be obtained from all participants previously to any data collection. Additionally, oral consent will be obtained at the beginning of the focus groups.

Ethical approval was obtained by the Ethical Committees of all the involved institutions – NOVA Medical School (ref. 73/2024/CEFCM; ref. 175/2024/CEFCM), i3S (ref. N10/CECRI/2024), Unidade Local de Saúde São João (ref. 265–23), University Hospital of Bellvitge (ref. PR075/24), University Hospital of Girona (ref. 2024.137), University Hospital Vall d’Hebron (ref. PR (AG)330/2023), CHU-Rouen (ref. 2025/0043/OB), RADBOUDUMC (ref. 2024–17792), Haukeland University Hospital (ref. 757236; ref. 789646; ref. 850836) and University Hospital Bonn (ref. 281/23-EP).

## Data availability statement

### Underlying data

No data are associated with this article.

### Extended data

OSF: Behavioural analysis of the referral and uptake of care pathways for RTRS, DOI: https://doi.org/10.17605/OSF.IO/KRQFA ^32^

The final surveys for patients and healthcare professionals, and the topic guides for patients, social networks and healthcare professionals are available at Open Science Framework ( https://doi.org/10.17605/OSF.IO/KRQFA).

This project contains the following extended data:

• Focus groups topic guides.pdf
• Survey clinicians.pdf
• Survey patients.pdf

All data are available on Open Science Framework.

Data are available under the terms of the Creative Commons Attribution 4.0 International license (CC-BY 4.0).

The final survey for patients is available at the Open Science Framework repository: https://doi.org/10.17605/OSF.IO/KRQFA

The final survey for healthcare professionals is available at the Open Science Framework repository: https://doi.org/10.17605/OSF.IO/KRQFA

The topic guides for the focus groups with patients, social networks and healthcare professionals are available at the Open Science Framework repository: https://doi.org/10.17605/OSF.IO/KRQFA

### Reporting guidelines

This study protocol followed the guidelines for pre-registry at Open Science Framework and publication in Open Research Europe. Resulting studies will be following the Consolidated Criteria for Reporting Qualitative Research (COREQ), ^33^ the Strengthening the Reporting of Observational Studies in Epidemiology (STROBE), ^34^ and the Good Reporting of A Mixed Methods Study (GRAMMS) ^35^ guidelines.
