# Supplementary material for: Behavioural analysis of referral and uptake of care pathways for rare tumour risk syndromes: A cross-cultural mixed-methods study protocol within the PREVENTABLE project
Source: Open Res Eur. 2026 Jul 6;5:108. Originally published 2025 Apr 15. [Version 2] doi: 10.12688/openreseurope.19954.2 (PMC13560810; doi:10.12688/openreseurope.19954.2)
Supplement: Peer review history

# Reviewer report: Reviewer response for version 2

**DOI:** 10.21956/openreseurope.26468.r77374

**Published:** 2026-08-20

**Authors:** Stuthi Perimbeti

**Affiliations:** 1 Penn State Cancer Institute, Hershey, USA

The authors have taken on a very rare and challenging research question, and they have addressed the prior reviewer comments thoroughly. There is a notable lack of published data in this area, particularly regarding the behavioral factors influencing healthcare professionals and the barriers affecting referral pathways and access to care. The development of the survey instruments and focus group discussion guides appears to be well thought out and methodologically sound. The findings are highly informative and have the potential to be applied to other clinical areas facing similar challenges. Overall, this work makes a valuable contribution to an underexplored field.

Is the study design appropriate for the research question?

Yes

Are sufficient details of the methods provided to allow replication by others?

Yes

Is the rationale for, and objectives of, the study clearly described?

Yes

Are the datasets clearly presented in a useable and accessible format?

Yes

Reviewer Expertise:

Genitourinary malignancies

I confirm that I have read this submission and believe that I have an appropriate level of expertise to confirm that it is of an acceptable scientific standard.

# Reviewer report: Reviewer response for version 2

**DOI:** 10.21956/openreseurope.26468.r76965

**Published:** 2026-07-10

**Authors:** Saskia F A Duijts

**Affiliations:** 1 Medical Psychology, Amsterdam University Medical Center, Amsterdam, The Netherlands

I would like to thank the authors for responding to my comments and concerns. I have reviewed the responses carefully, and consider them thorough, and well-reasoned. The concerns have been addressed in a satisfactory manner. In my view, the manuscript has been strengthened as a result of the revisions. I have no further comments and support the manuscript in its revised form.

Is the study design appropriate for the research question?

Yes

Are sufficient details of the methods provided to allow replication by others?

Yes

Is the rationale for, and objectives of, the study clearly described?

Yes

Are the datasets clearly presented in a useable and accessible format?

Yes

Reviewer Expertise:

Psychosocial oncology; rare cancers

I confirm that I have read this submission and believe that I have an appropriate level of expertise to confirm that it is of an acceptable scientific standard.

# Reviewer report: Reviewer response for version 1

**DOI:** 10.21956/openreseurope.21585.r54335

**Published:** 2025-06-05

**Authors:** Saskia F A Duijts

**Affiliations:** 1 Medical Psychology, Amsterdam University Medical Center, Amsterdam, The Netherlands

ABSTRACT (and plain language summary)

 - The authors state as a gap / problem that 'the behavioural factors influencing this decision making process and best communication practices are unknown'. Then: the aim of PREVENTABLE is to assess the clinical, social and economic impact of RTRS-care pathways. Whilke the gap / problem is clear, and the aim is clear: the combination of the two is less clear. However, this becomes clear when looking at the specifc objective for THIS study. To prevent confusion, replace the overall aim of PREVENTABLE to the objective section. First state the aim of the current study there, and then that the study is part of ... and so on.

 - Not clear where (n=100) is referring to. Or do the authors actually mean 100 clinical teams? The same goes for n=290. Since this is places at the end of the sentence, it is not fully clear where it relates to. I suppose patients? Yet, this sentence is about patients' adoption. Or do the authors refer to 290 care pathways? Anyways, it is unclear (when reading the abstract only). 

 - In the plain language summary, the authors talk about social networks. This has nog been mentioned in the abstract. 

 BACKGROUND

 - It increases readability when sentences are less long. First sentence is 6 lines! Please place a period (.) after / begin new sentence: ... up to 100%. Since these syndromes follow... 

 - The authors state that... ultimately, they aim to develop person-centred guidelines to improve communication and recommendation of prevention-related care pathways for RTRS. It is unclear / not sufficienlty addressed throughout the paper if this concerns guidelines for individual countries, nor what gathering data in several countries (with - supposingly - different care pathways regarding RTRS) implies and how this will be taken into account addressing this aim. 

 PROTOCOL

 - One of the surveys wll be for clinical teams managing RTRS. Will specific persons in a team be invited to complete the survey? Will teams be invited to complete the survey as a team? Probably, this will be explained, yet, at this point, it raises questions. 

 - Split the inclusion criteria in three groups (clinical teams, individuals, social network). - Here, it becomes clear that the number of persons to be included is 100 professionals and 290 patients. Not clear in the abstract. Also, what about the number of persons from the social networks of patients? Or wil they only participate in the FG?

 - The surveys are developed in English? What about the translation to Portugese, Spanish and so on. And cross-cultural translation and validation of the survey in each country to make sure similar concepts will be measured? Note: later on it becomes clear that translation to each PREVENTABLE language has taken place. Throughout the paper, some issues raise questions yet are answered later on in the paper. While it is good that such issues become clear later on, try to avoid confusion at the first place. 

 - The authors talk about the survey for clinical teams and for healthcare professionals. Maybe better to explain only once that the survey will be distributed amongst healthcare professionals of clinical teams, and then stick to healthcare professionals. 

 - Regarding data analysis, the authors state that they focus on behavioural analysis of referral and uptake of care pathways. Both the behavioural analyses and how they define care pathways need more explanation.

Is the study design appropriate for the research question?

Yes

Are sufficient details of the methods provided to allow replication by others?

Yes

Is the rationale for, and objectives of, the study clearly described?

Yes

Are the datasets clearly presented in a useable and accessible format?

Yes

Reviewer Expertise:

Psychosocial oncology; rare cancers

I confirm that I have read this submission and believe that I have an appropriate level of expertise to confirm that it is of an acceptable scientific standard, however I have significant reservations, as outlined above.

# Response

**Published:** 2026-06-26

**Authors:** Maiara Eliza Moreto

We sincerely thank you for your time, thorough reading, and constructive feedback on our manuscript. Your suggestions have greatly improved the structure, clarity, and readability of our work. Please find below our point-by-point responses.

**1. The authors state as a gap/problem that 'the behavioural factors influencing this decision-making process and best communication practices are unknown'. The aim of PREVENTABLE is to assess the clinical, social and economic impact of RTRS-care pathways. While the gap and the aim are clear, the combination of the two is less clear. To prevent confusion, replace the overall aim of PREVENTABLE to the objective section. First state the aim of the current study there, and then that the study is part of... and so on.**

**Response:** We agree with this structural suggestion. We have restructured the abstract to separate the specific study aim from the broader project context. The overall aim of PREVENTABLE has been moved to the Objective section. The abstract now first states the aim of the current study, followed by a clarification that it is conducted within the framework of the PREVENTABLE project.

**2.** **Not clear where (n=100) is referring to. Or do the authors actually mean 100 clinical teams? The same goes for n=290. Since this is places at the end of the sentence, it is not fully clear where it relates to. I suppose patients? Yet, this sentence is about patients' adoption. Or do the authors refer to 290 care pathways? Anyways, it is unclear (when reading the abstract only).**

**Response:** Thank you for pointing this out. We agree that the abstract text needed to specify the exact units being sampled. The abstract has been revised to make explicit that *n*=100 refers to members of clinical teams and *n*=290 refers to individual patients with RTRS.

**3. In the plain language summary, the authors talk about social networks. This has not been mentioned in the abstract.**

**Response:** We thank the reviewer for this observation. Social networks are mentioned in the abstracs, specifically in the Objectives and Methods sections. The restructuring described in our response to Comment 1 has made the integration of social networks much more explicit and highly visible to the reader in the revised abstract.

**4. It increases readability when sentences are less long. First sentence is 6 lines! Please place a period (.) after / begin new sentence: ...up to 100%. Since these syndromes follow...**

**Response:** We completely agree with this assessment. The opening sentence of the Background section has been divided into two shorter, clearer sentences, exactly as suggested: 

**5.** **The authors state that... ultimately, they aim to develop person-centred guidelines to improve communication and recommendation of prevention-related care pathways for RTRS. It is unclear / not sufficienlty addressed throughout the paper if this concerns guidelines for individual countries, nor what gathering data in several countries (with - supposingly - different care pathways regarding RTRS) implies and how this will be taken into account addressing this aim.**

**Response:** This is an important point regarding our methodology, and we thank the reviewer for raising it. We have clarified throughout the manuscript that our mixed-methods design allows for cross-cultural comparison of COM-B domains, and that country-level differences will be explicitly examined during data analysis. The Objectives section now explicitly frames the study as a multi-country investigation. The Data Analysis section states that inferential statistics will be used to examine whether COM-B domains differ by country, and that qualitative analysis will compare variations in how COM-B domains manifest across regions. Finally, the reference to person-centered guidelines has been moved to the Conclusion section where we clarify that these guidelines will draw directly on the integrated cross-cultural findings.

**6.** **One of the surveys will be for clinical teams managing RTRS. Will specific persons in a team be invited to complete the survey? Will teams be invited to complete the survey as a team? Probably, this will be explained, yet, at this point, it raises questions.**

**Response:** The survey was directed to members of clinical teams with expertise in RTRS, rather than at entire clinical teams completing a single survey collectively. Each eligible professional was invited to complete the survey individually and anonymously. To make this clear from the outset, we have revised the Design section to specify that the survey targets individual members of clinical teams rather than teams as a collective unit.

**7.** **Split the inclusion criteria in three groups (clinical teams, individuals, social network). Here, it becomes clear that the number of persons to be included is 100 professionals and 290 patients. Not clear in the abstract. Also, what about the number of persons from the social networks of patients? Or wil they only participate in the FG?**

**Response:** We appreciate this suggestion to improve the structural layout of our participant descriptions. We have reorganized the Population section to present the inclusion criteria separately for each stakeholder group and each study phase (surveys and focus groups). This restructuring clarifies that the social network participants are exclusively included in the focus group phase. We have also detailed their sample size based on qualitative standards (~6 participants per group), yielding 18 focus groups in total across the study (108 participants; 36 per target group).

**8.** **The surveys are developed in English? What about the translation to Portugese, Spanish and so on. And cross-cultural translation and validation of the survey in each country to make sure similar concepts will be measured? Note: later on it becomes clear that translation to each PREVENTABLE language has taken place. Throughout the paper, some issues raise questions yet are answered later on in the paper. While it is good that such issues become clear later on, try to avoid confusion at the first place.**

**Response:** We thank the reviewer for this feedback and for the broader observation regarding text flow. We acknowledge that introducing operational details late in the text can raise questions, and we have adjusted the sequencing to address this. The survey development subsection has been revised to immediately clarify that surveys were developed in English and subsequently translated into all six PREVENTABLE languages using the European Commission's eTranslation service. We now explicitly specify that a forward-backward translation protocol was utilized, and that PREVENTABLE clinical partners (i.e., bilingual experts with deep knowledge of both RTRS and local healthcare contexts) reviewed all translations to ensure cultural and conceptual adequacy.

**9.** **The authors talk about the survey for clinical teams and for healthcare professionals. Maybe better to explain only once that the survey will be distributed amongst healthcare professionals of clinical teams, and then stick to healthcare professionals.**

**Response:** We agree that using multiple terms interchangeably created unnecessary ambiguity. We have standardized our terminology across all sections of the manuscript. The phrase *"members of clinical teams"* is now used consistently.

**10. Regarding data analysis, the authors state that they focus on behavioural analysis of referral and uptake of care pathways. Both the behavioural analyses and how they define care pathways need more explanation.**

**Response:** We agree that foundational concepts must be explicitly introduced early on. We have added an explicit definition of "care pathways" in the Background section and incorporated a dedicated paragraph defining behavioural analysis. These modifications ensure both core concepts are explicitly defined and contextualized before they are applied in the Methods and Data Analysis sections.

# Reviewer report: Reviewer response for version 1

**DOI:** 10.21956/openreseurope.21585.r53345

**Published:** 2025-05-19

**Authors:** Nickola Pallin

**Affiliations:** 1 Trinity College Dublin, Dublin, Ireland

Thank you for the opportunity to review this study protocol. I think an appropriate plan has been developed for the study. I left some comments below which I hope will be helpful to the authors.

 The manuscript refers to 'care pathways' as the focus of the study and it would be helpful to clarify what is meant by this term in the research context.

 Consider expanding on what the behavioural factors being explored are influencing or what the ‘target behaviour’ is. This would improve clarity as it is not entirely clear whether the focus is on the care pathways themselves, the recommendation of those pathways by HCPs, communication, decision-making by those with RTRS, or the interaction between these elements. At times this distinction is blurred.

 It would strengthen the manuscript by explaining the intervention or practice being studied (e.g., the RTS care pathways, communication strategies, or decision-making processes) and the behaviour being investigated (e.g., referral of the pathway or patient uptake).

 The sentence “Furthermore, gaps still persist in understanding the various factors influencing decision-making in RTRS, such as, preferences of individuals with RTRS, healthcare professionals recommendations, and the best practices for communication and emotional support in RTRS care” includes several important points, but they are a bit crowded and the authors might consider breaking this up. In addition, the sentence ‘best practices for communication and emotional support in RTRS care’ feels loosely connected to the rest of the list which makes the message hard to follow. The authors might consider clarifying how this sentence relates to decision-making and care pathways.

 The manuscript proposes the use of the COM-B model. It would be helpful if the authors explain why COM-B has been selected instead of other determinant frameworks as the focus of the study is on identifying barriers and enables.

 The paper seems to equate behavioural factors with barriers and facilitators, but this could be clarified. Not all barriers and facilitators may be behavioural, for example, resources or guidelines and policies may influence opportunity or capability without being behavioural themselves. Clarifying how these factors (barriers and facilitators) are behavioural factors and influence the recommendation and uptake of care pathways would strengthen the paper.

 Clarifying what is meant by behavioural analysis would be helpful.

 Aim 3 - it is unclear whether recommendation refers to those made by the clinical team or those made by the social network.

 I was also unsure if the study is exploring the perception of social network members on the barriers and facilitators that individuals with RTRS experience or if it is the barriers and facilitators that the social network experience. This clarification will help the reader understand aim 3 and objective iii (in the abstract), as well as the purpose of including social networks and family in this study.

 The paper mentions sample sizes for healthcare professionals and patients, but I cannot seem to see a sample size provided for the social networks.

 Is there a separate survey for social networks? this should be clarified in the methods section.

 Was there only a sample size calculation for the RTRS sample? I was wondering why this is.

 Will inferential statistics be conducted for all the surveys?

 Using inferential statistics what categories will be compared to the COM-B domains?

 The authors note that they will use triangulation to integrate survey and focus group data. The surveys are designed to identify barriers and facilitators, and it seems that the focus groups are described more generally in terms of exploring factors and challenges. For greater coherence, the authors could clarify how the focus group findings will directly address the overall study aims.

 The authors could expand on how they plan to handle the analysis and integration of data across the three groups (HCPs, patients, and social networks) for each of the surveys and focus groups. The authors could also clarify how all these elements will be integrated within the mixed methods design and joint display and narrative synthesis. A figure illustrating these steps might also be useful here. 

 The last two sentences of the data analysis where the authors state “This integration of the results of the focus groups and surveys will inform the development of guidelines on how the care pathways for RTRS….” I suggest moving this statement to the discussion, where the implications of the study findings for guideline development could be discussed.

Is the study design appropriate for the research question?

Yes

Are sufficient details of the methods provided to allow replication by others?

Partly

Is the rationale for, and objectives of, the study clearly described?

Partly

Are the datasets clearly presented in a useable and accessible format?

Yes

Reviewer Expertise:

Implementation Science, Oncology

I confirm that I have read this submission and believe that I have an appropriate level of expertise to confirm that it is of an acceptable scientific standard, however I have significant reservations, as outlined above.

# Response

**Published:** 2026-06-26

**Authors:** Maiara Eliza Moreto

We sincerely thank you for your time and constructive feedback on our manuscript. Your comments have improved the clarity and quality of the paper. Please find below our point-by-point responses. 

**1. The manuscript refers to 'care pathways' as the focus of the study and it would be helpful to clarify what is meant by this term in the research context.**

**Response: **We thank the reviewer for this observation. We have now added an explicit definition in the second paragraph of the Background section. The revised text clarifies that care pathways refer to sequences of care options involving multiple decisions, including primary prevention (e.g., risk-reducing surgery, chemoprevention, changes in diet), surveillance (e.g., earlier, more frequent mammogram/colonoscopy), and treatment for cancer or premalignant conditions.

**2.** **The sentence "Furthermore, gaps still persist in understanding the various factors influencing decision-making in RTRS, such as, preferences of individuals with RTRS, healthcare professionals recommendations, and the best practices for communication and emotional support in RTRS care" includes several important points, but they are a bit crowded. The sentence 'best practices for communication and emotional support in RTRS care' feels loosely connected to the rest of the list** **which makes the message hard to follow. The authors might consider clarifying how this sentence relates to decision-making and care pathways.**

**Response: **We agree. The original sentence was dense and the connection between communication/emotional support and decision-making was insufficiently explicit. We have broken up the sentence and now connect communication challenges and the emotionally charged nature of decision-making in RTRS explicitly to the gap in care.

**3.** **The manuscript proposes the use of the COM-B model. It would be helpful if the authors explain why COM-B has been selected instead of other determinant frameworks, as the focus of the study is on identifying barriers and enables.**

**Response: **We thank the reviewer for this important suggestion. A justification for the selection of the COM-B model has been added following the explanation of the model in the third paragraph of the Background section. We highlight the model's capacity to capture both internal (psychological capability and motivation) and external (physical and social opportunities) determinants of behavior, enabling systematic identification of barriers and facilitators. We additionally note the complementary use of the Theoretical Domains Framework (TDF) for the qualitative component.

**4.** **Consider expanding on what the behavioural factors being explored are influencing or what the ‘target behaviour’ is. This would improve clarity as it is not entirely clear whether the focus is on the care pathways themselves, the recommendation of those pathways by HCPs, communication, decision-making by those with RTRS, or the interaction between these elements. At times this distinction is blurred.**

**Response: **We thank the reviewer for pointing this out. The two distinct target behaviours of the study: (i) referral and recommendation of RTRS care pathways by clinical teams, and (ii) uptake of recommended care by patients, are now clearly stated and distinguished in the Background section. The text also clarifies that the study investigates individual, social, and environmental determinants of each behaviour.

**5.** **It would strengthen the manuscript by explaining the intervention or practice being studied (e.g., the RTS care pathways, communication strategies, or decision-making processes) and the behaviour being investigated (e.g., referral of the pathway or patient uptake).**

**Response: **In line with the previous comment, we have provided a clearer account of both the clinical context (RTRS care pathways) and the specific behaviours under investigation in the final paragraph of the Background section. The two target behaviours are explicitly named and distinguished from the care pathways themselves, which are the object of the behavioural analysis rather than the behaviour per se.

**6.** **The paper seems to equate behavioural factors with barriers and facilitators, but this could be clarified. Not all barriers and facilitators may be behavioural, for example, resources or guidelines and policies may influence opportunity or capability without being behavioural themselves. Clarifying how these factors (barriers and facilitators) are behavioural factors and influence the recommendation and uptake of care pathways would strengthen the paper.**

**Response: **The reviewer raises a valid conceptual point. We have revised the language in the final paragraph of the Background section to refer more precisely to "individual, social, and environmental determinants" rather than using "behavioural factors" as an umbrella term for all barriers and facilitators. This avoids conflating the behavioural analysis framework with the full range of determinants captured under the COM-B model, which includes non-behavioural factors such as environmental resources and social norms.

**7.** **Clarifying what is meant by behavioural analysis would be helpful.**

**Response: **We have added a dedicated paragraph in the Background section defining behavioural analysis in the context of this study. 

**8.** **Aim 3 — it is unclear whether recommendation refers to those made by the clinical team or those made by the social network.**

**Response: **We thank the reviewer for this observation. Aim 3 has been rewritten to make clear that the focus is on the barriers and facilitators experienced by the social network itself — specifically, how they provide support for, engage in, and influence the uptake of care pathways — not on recommendations made by the social network. 

**9.** **I was also unsure if the study is exploring the perception of social network members on the barriers and facilitators that individuals with RTRS experience or if it is the barriers and facilitators that the social network experience. This clarification will help the reader understand aim 3 and objective iii (in the abstract), as well as the purpose of including social networks and family in this study.**

**Response: **We clarify that this study explores the barriers and facilitators experienced by the social network when providing support for and engaging in the uptake of the recommended care pathway for RTRS. The third aim has been revised accordingly. This now clearly distinguishes the perspective of the social network from that of the patients themselves.

 1 **0.** **The authors note that they will use triangulation to integrate survey and focus group data. The surveys are designed to identify barriers and facilitators, and it seems that the focus groups are described more generally in terms of exploring factors and challenges. For greater coherence, the authors could clarify how the focus group findings will directly address the overall study aims.**

**Response: **We agree that this needed greater clarity. The Design section now explicitly states that focus groups serve to explore participants' experiences, perceptions, and contextual factors related to the barriers and enablers identified in the survey phase, thereby providing a broader understanding of the quantitative findings. 

**11.** **Is there a separate survey for social networks? This should be clarified in the methods section.**

**Response: **The social network was not included in the survey phase. To make this explicit, we have added a clarifying sentence in the Design section stating that members of the social network are only included in Step 2 (focus groups) as an additional source and are not part of the survey phase.

**12.** **The paper mentions sample sizes for healthcare professionals and patients, but I cannot seem to see a sample size provided for the social networks.**

**Response: **As noted above, the social network was not included in the survey phase. Sample size was determined only for the two survey target groups (members of clinical teams: n=100; individuals with RTRS: n=290). Social network members participate only in the focus groups, where sample size follows established focus group recommendations (approximately six participants per group). This is now clearly stated in the Population section.

**13.** **Was there only a sample size calculation for the RTRS sample? I was wondering why this is.**

**Response: **A sample size calculation was conducted for both target groups. For individuals with RTRS, the calculation was based on the total number of cases identified within the PREVENTABLE consortium (n=1177), using a 95% confidence interval and 5% margin of error. For members of clinical teams, the sample size was estimated based on the number of healthcare professionals with expertise in the RTRS included in the project and the composition of the participating teams across countries. This distinction is now explicitly stated in the Population section.

**14.** **Will inferential statistics be conducted for all the surveys?**

**Response: **Yes, inferential statistics will be applied to the survey data for both target groups (members of clinical teams and patients with RTRS). The Data Analysis section outlines this approach. Focus group data, by contrast, will be analysed using thematic analysis. Both phases are structured around the COM-B framework, which allows for coherent integration of findings across methods.

**15.** **Using inferential statistics, what categories will be compared to the COM-B domains?**

**Response: **We have added more detail on this point in the Data Analysis section. Inferential statistics will be used to explore: (i) comparisons between the different care pathways, (ii) within-domain comparisons of more granular constructs, and (iii) comparisons of total scores across COM-B domains. In addition, we will examine whether COM-B domains differ by syndrome, clinical specialty, and country.

**16.** **The authors could expand on how they plan to handle the analysis and integration of data across the three groups (HCPs, patients, and social networks) for each of the surveys and focus groups. The authors could also clarify how all these elements will be integrated within the mixed methods design and joint display and narrative synthesis. A figure illustrating these steps might also be useful here.**

**Response: **We have clarified in the Data Analysis section that data will be integrated within each stakeholder group separately, with findings from different groups reported and interpreted independently. Triangulation within each group will be achieved through narrative integration using a contiguous approach and through joint displays, allowing side-by-side presentation of findings.

**17.** **The last two sentences of the data analysis where the authors state “This integration of the results of the focus groups and surveys will inform the development of guidelines on how the care pathways for RTRS….” I suggest moving this statement to the discussion, where the implications of the study findings for guideline development could be discussed.**

**Response: **We agree with this suggestion. The relevant sentences have been moved to the Conclusion section, where they more appropriately convey the practical implications of the study findings for guideline development.
